# Analysis of risk factors for calf muscular vein thrombosis in elderly patients with acute exacerbation of chronic obstructive pulmonary disease

**DOI:** 10.3389/fcvm.2026.1742275

**Published:** 2026-01-30

**Authors:** Xiaolong Li, Shuhao Xu, Xin Wang, Yuanyuan Liu, Chunfang Zeng, Yang Hu, Rongli Wang

**Affiliations:** 1 Pulmonary and Critical Care Medicine, Deyang People’s Hospital, Deyang, Sichuan, China; 2 Department of Stomatology, Deyang People’s Hospital, Deyang, Sichuan, China; 3 Department of Practical Training, Sichuan Nursing Vocational College, Deyang, Sichuan, China; 4 The Affiliated Hospital of Southwest Medical University, Luzhou, Sichuan, China

**Keywords:** acute exacerbation, calf circumference, calf muscular vein thrombosis, chronic obstructive pulmonarydisease, D-dimer, RBC, risk factors

## Abstract

**Objective:**

This study aimed to identify the independent risk factors for calf muscular vein thrombosis (CMVT) in elderly patients experiencing an acute exacerbation of chronic obstructive pulmonary disease (AECOPD).

**Methods:**

A retrospective study was conducted involving 128 elderly patients (age ≥60 years) with AECOPD. Patients were categorized into CMVT and non-CMVT groups based on lower extremity venous color Doppler ultrasound findings. Clinical characteristics and laboratory parameters were compared between the groups. Statistically significant variables from univariate analysis were incorporated into a multivariate logistic regression analysis to identify independent risk factors. The predictive performance of these factors was evaluated using receiver operating characteristic (ROC) curve analysis.

**Results:**

Multivariate logistic regression identified reduced calf circumference [Odds Ratio (OR) = 0.25, 95% Confidence Interval (CI): 0.1–0.59], elevated red blood cell (RBC) count (OR = 19.85, 95% CI: 1.08–363.96), and elevated D-dimer level (OR = 1.84, 95% CI: 1.13–3.01) as independent risk factors for CMVT. ROC curve analysis demonstrated good predictive performance for these factors, with areas under the curve (AUC) of 0.986 for calf circumference, 0.788 for RBC count, and 0.976 for D-dimer.

**Conclusion:**

Reduced calf circumference, elevated RBC count, and elevated D-dimer level are significant independent risk factors for CMVT in elderly AECOPD patients. Monitoring these indicators could aid clinicians in the early identification and prevention of CMVT in this vulnerable population.

## 1 Introduction

Chronic Obstructive Pulmonary Disease (COPD) is a common, preventable, and treatable chronic airway disease characterized by persistent airflow limitation and corresponding respiratory symptoms. The primary clinical manifestations of COPD include chronic cough, sputum production, and dyspnea. According to the Global Burden of Disease study, COPD is the fifth leading cause of death in China. With the accelerating aging of the population, the prevalence of COPD is projected to continue rising over the next 40 years, and the annual number of deaths due to COPD and its related complications is expected to exceed 5 million in the coming decades (1, 2).

Calf muscular vein thrombosis (CMVT) refers to thrombosis that originates and is confined to the venous plexus of the gastrocnemius and soleus muscles, belonging to the category of peripheral deep vein thrombosis (DVT). The soleal and gastrocnemius venous plexuses are the most frequently involved sites, primarily attributable to factors such as their narrow lumina, numerous tributaries, sparse venous valves, and lack of deep fascial envelopment (3). Due to the minimal impact of CMVT on venous return and the weak systemic inflammatory response it elicits, affected patients are often asymptomatic, leading to frequent underdiagnosis by clinicians (4).

In clinical practice, CMVT is not uncommonly among elderly patients experiencing acute exacerbation of COPD (AECOPD). Studies have reported a higher incidence of venous thromboembolism (VTE) in patients with COPD compared to those without COPD, with approximately 10.5% of AECOPD patients presenting with DVT (5–7). Advanced age has been identified as a risk factor for VTE, with the risk increasing correspondingly with age (8, 9). However, the exact incidence of CMVT in elderly patients with AECOPD remains unclear. Failure to identify CMVT early in elderly patients with AECOPD may lead to proximal thrombus extension and development to DVT, potentially even triggering fatal pulmonary embolism (3). Moreover, research specifically focused on CMVT in elderly patients with AECOPD is relatively limited. This study aimed to retrospectively analyze the main risk factors for CMVT in this population to assist clinicians in the early and effective identification of high-risk patients, thereby providing a basis for early thromboprophylaxis strategies.

## 2 Subjects and Methods

### 2.1 Subjects

**Figure 1:**
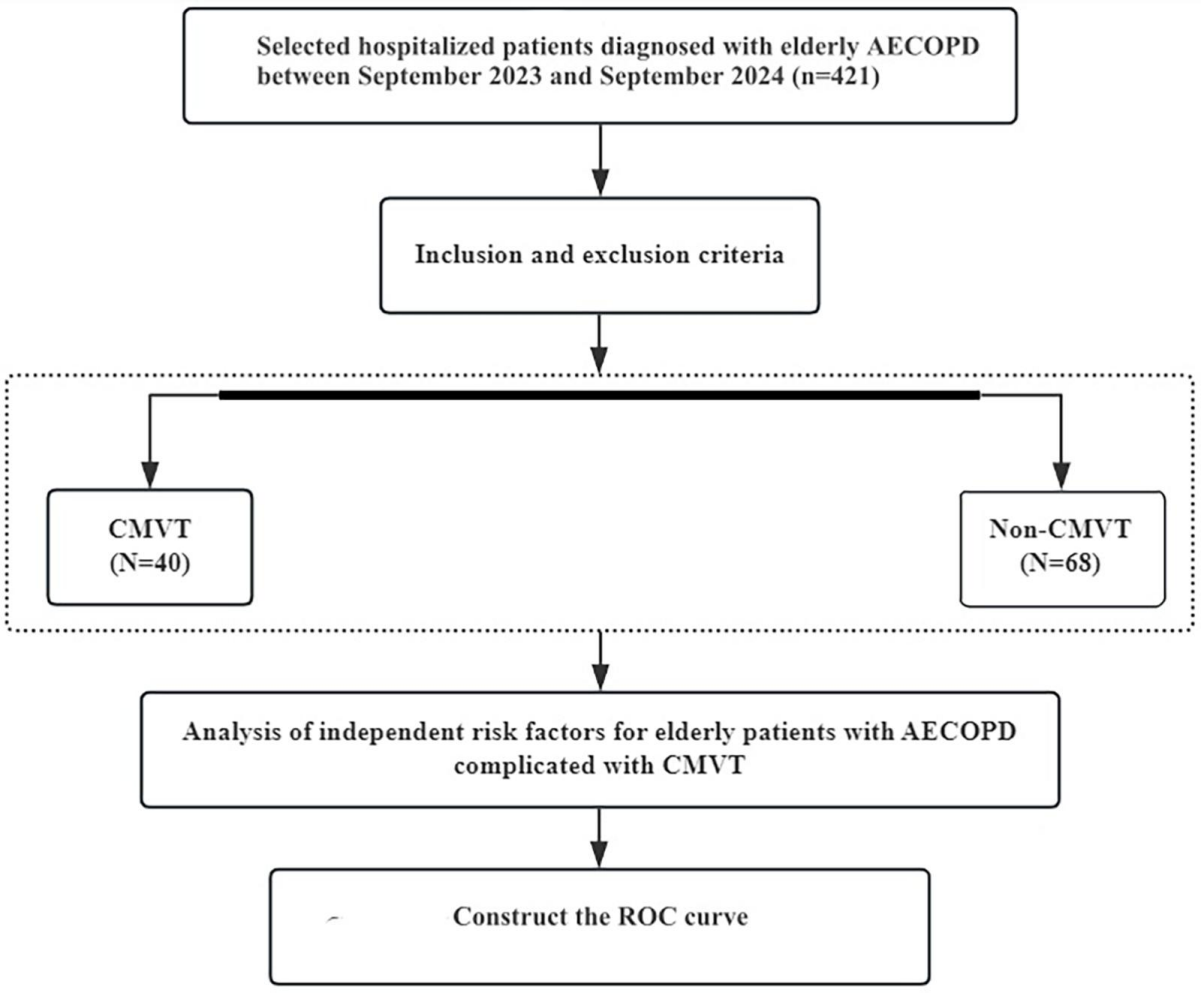
Flow chart describing the research method.

A total of 421 patients with AECOPD who were treated in the Deyang People's Hospital between September 2023 and September 2024 were included in this retrospective study. Inclusion criteria: (1) Age ≥60 years. (2) Discharge diagnosis of AECOPD, consistent with the diagnostic criteria established in the Global Initiative for Chronic Obstructive Lung Disease (GOLD) 2023 Guidelines (10). This required the presence of symptoms such as chronic cough, sputum production, dyspnea, wheezing, or chest tightness, along with pulmonary function tests confirming persistent airflow limitation [post-bronchodilator forced expiratory volume in the 1st second/forced vital capacity (FEV_1_/FVC) < 70%]. Exclusion criteria: (1) Age <60 years. (2) Incomplete medical records. (3) Missing essential laboratory data or absence of relevant imaging studies. (4) Presence of DVT in locations other than the calf muscular veins; (5) Coexisting diagnoses of bronchial asthma, bronchiectasis, pulmonary tuberculosis, diffuse panbronchiolitis, lung cancer, or pulmonary embolism. Finally, 128 elderly patients with AECOPD were enrolled in the study, among whom 40 patients were diagnosed with concomitant CMVT, and 88 patients were without CMVT (Figure 1). Technique and diagnostic criteria for lower limb venous ultrasonography: (1) The primary criterion was the incomplete or absent compressibility of the venous lumen under transducer pressure. (2) Intraluminal Filling Defect: Visualization of an echogenic or hypoechoic filling defect within the vein. (3) Absence of Spontaneous Flow: Lack of spontaneous color Doppler or spectral Doppler flow signal within the affected venous segment. (4) Loss of Flow Phasicity: Absence of normal respiratory phasicity in Doppler waveform in veins proximal to the thrombus (if assessable). This study was conducted in accordance with the principles of the Declaration of Helsinki and was approved by the Ethics Committee of Deyang People's Hospital.

### 2.2 Data collection

The collected data included:

1. Clinical data: age, gender, history of diabetes mellitus (DM), hypertension, coronary heart disease (CHD), cerebral infarction, atrial fibrillation (AF), heart failure (HF), alcohol consumption, smoking, previous history of DVT, duration of hospital stay, history of mechanical ventilation, receipt of prophylactic anticoagulation (during hospitalization), and calf circumference (measured at the maximum circumference of the non-dominant lower limb).
2. Laboratory data at admission: white blood cell count (WBC), neutrophil count (NEUT), red blood cell count (RBC), platelet count (PLT), D-dimer, high-sensitivity C-reactive protein (hs-CRP), aspartate aminotransferase (AST), alanine aminotransferase (ALT), albumin (ALB), Creatinine, pH, arterial partial pressure of oxygen (PaO_2_), arterial partial pressure of carbon dioxide (PaCO_2_), serum sodium, serum potassium, blood lactate, blood uric acid, prothrombin time (PT), international normalized ratio (INR), activated partial thromboplastin time (APTT), fibrinogen (FIB), and thrombin time (TT).
3. Examination data: pulmonary function grade, severity of COPD exacerbation, and presence or absence of sputum production.
4. Group definitions: Elderly COPD patients with concurrent CMVT were classified as the CMVT group, while those without CMVT were the non-CMVT group.

### 2.3 Statistical analysis

Statistical analysis was performed using Statistical Package for the Social Sciences (SPSS) software (version 26.0). Continuous variables conforming to a normal distribution are presented as mean ± standard deviation (SD), and comparisons between groups were conducted using the independent samples *t*-test. Categorical data are presented as numbers (percentages), and intergroup comparisons were performed using the chi-square (χ^2^) test. Continuous variables with a non-normal distribution are presented as median and interquartile range [M (P25, P75)], and the Mann–Whitney *U*-test was used for group comparisons. Statistically significant variables were identified through univariate analysis.

Variables that showed significant in the univariate analysis were included in a multivariate logistic regression analysis to identify independent risk factors, and their odds ratios (OR) with 95% confidence intervals (CI) were calculated. The predictive performance of the independent risk factors was evaluated using receiver operating characteristic (ROC) curve analysis, and the area under the curve (AUC) was calculated.

## 3 Results

### 3.1 Comparison of clinical characteristics

Compared with the non-CMVT group, the CMVT group demonstrated a significantly smaller calf circumference (*P* < 0.05, Table 1). However, no statistically significant differences were observed between the two groups regarding age, gender, history of DM, hypertension, CHD, cerebral infarction, alcohol consumption, smoking, prolonged immobilization, previous history of DVT, duration of hospital stay, history of mechanical ventilation, receipt of prophylactic anticoagulation (during hospitalization) or severity of COPD exacerbation (*P* > 0.05, Table 2).

**Table 1:** Laboratory data at admission.

Laboratory Data	N-CMVT (*n* = 68)	CMVT (*n* = 40)	*T/Z*	*p*
WBC (×10^9^)	8.44 ± 3.45	9.62 ± 4.41	−1.493	0.141
NEU (×10^9^)	6.61 ± 3.35	8.93 ± 10.38	−1.382	0.174
RBC (×10^12^)	4.2 ± 0.84	5.08 ± 0.85*	−5.419	<0.001
pH	7.38 ± 0.06	7.4 ± 0.08	−1.019	0.312
PaCO2 (mmHg)	50.84 ± 14.44	48.56 ± 15.61	0.806	0.422
PaO2 (mmHg)	89.08 ± 15.9	76.86 ± 12.59	0.778	0.003
PLT (×10^9^)	194.12 ± 75.96	180.85 ± 75.01	0.92	0.359
hs-CRP (mg/L)	34.2 ± 44.89	45.68 ± 56.25	−1.123	0.266
PT (s)	12.56 ± 2.56	12.38 ± 1.01	0.582	0.562
ALT (U/L)	24 (18,32)	23.5 (19.75,36)	−0.702	0.483
AST (U/L)	26 (19.75,33.5)	30 (22,37)	−1.615	0.106
INR	1.09 ± 0.24	1.06 ± 0.09	0.746	0.457
APTT (s)	26.54 ± 2.31	27.55 ± 3.43	−1.695	0.096
FIB (g/L)	4.47 ± 1.71	4.5 ± 1.81	−0.088	0.93
TT (s)	18.93 ± 1.48	18.69 ± 1.22	0.864	0.389
D-dimer (mg/L FEU)	1.43 ± 3.01	6.36 ± 2.39*	−9.132	<0.001
ALB (g/L)	38.82 ± 6.34	37.09 ± 4.79	1.704	0.091
Creatinine (mmol/L)	78.11 ± 34	77.03 ± 22.83	0.211	0.834
Blood Uric Acid (umol/L)	330.53 ± 115.32	312.52 ± 137.93	0.769	0.443
Serum Potassium (mmol/L)	3.76 ± 0.62	3.87 ± 0.48	−1.163	0.248
Serum Sodium (mmol/L)	137.46 ± 4.54	136.59 ± 3.64	1.064	0.289
Blood Lactate (mmol/L)	1.91 ± 0.74	1.75 ± 0.61	1.143	0.255

* Compared with N-CMVT, *p* < 0.05.

**Table 2:** Clinical characteristics.

Parameters	N-CMVT (*n* = 68)	CMVT (*n* = 40)	χ^2^/*t*	*p*
Age (year)	75.11 ± 9.02	73.78 ± 7.81	0.81	0.419
Calf circumference (cm)	33.3 ± 2.74	25.89 ± 2.43*	14.672	<0.001
Duration of hospital (d)	9.02 ± 2.36	8.93 ± 2.83	0.204	0.839
Gender (*n*)				
Female	31 (35.2%)	12 (30%)	0.337	0.562
Male	57 (64.8%)	28 (70%)		
Diabetes mellitus (*n*)				
No	74 (84.1%)	30 (75%)	1.492	0.222
Yes	14 (15.9%)	10 (25%)		
Hypertension (*n*)				
No	57 (64.8%)	24 (60%)	0.27	0.604
Yes	31 (35.2%)	16 (40%)		
CHD (*n*)				
No	81 (92%)	34 (85%)	0.823	0.364
Yes	7 (8%)	6 (15%)		
Cerebral infarction (*n*)				
No	85 (96.6%)	37 (92.5%)	0.318	0.573
Yes	3 (3.4%)	3 (7.5%)		
Atrial fibrillation (*n*)				
No	85 (96.6%)	38 (95%)	0	1
Yes	3 (3.4%)	2 (5%)		
Heart failure (*n*)				
No	83 (94.3%)	35 (87.5%)	0.955	0.329
Yes	5 (5.7%)	5 (12.5%)		
Alcohol consumption (*n*)				
No	54 (61.4%)	18 (45%)	2.992	0.084
Yes	34 (38.6%)	22 (55%)		
Smoking (*n*)				
No	40 (45.5%)	15 (37.5%)	0.71	0.399
Yes	7 (8%)	8 (20%)		
History of DVT (*n*)				
No	83 (94.3%)	35 (87.5%)	0.955	0.329
Yes	5 (5.7%)	5 (12.5%)		
Sputum (*n*)				
No	42 (47.7%)	17 (42.5%)	0.302	0.582
Yes	46 (52.3%)	23 (57.5%)		
Pulmonary function grade (*n*)				
1	3 (3.4%)	5 (12.5%)	3.894	0.273
2	21 (23.9%)	9 (22.5%)		
3	39 (44.3%)	16 (40%)		
4	25 (28.4%)	10 (25%)		
Severity of COPD exacerbation (*n*)				
Grade 1	23 (26.1%)	10 (25%)	0.198	0.906
Grade 2	36 (40.9%)	18 (45%)		
Grade 3	29 (33%)	12 (30%)		
History of mechanical ventilation (*n*)				
No	80 (90.9%)	36 (90%)	0	1
Yes	8 (9.1%)	4 (10%)		
Receiving prophylactic anticoagulation (during hospitalization) (*n*)				
No	82 (93.2%)	37 (92.5%)	0	1
Yes	6 (6.8%)	3 (7.5%)		

* Compared with N-CMVT, *p* < 0.05.

### 3.2 Comparison of laboratory parameters

Compared with the non-CMVT group, the CMVT group exhibited significantly higher RBC count and D-dimer levels (*P* < 0.05), along with a significantly lower PaO_2_ (*P* < 0.05). No statistically significant differences were found between the two groups in terms of WBC, NEUT, PLT, hs-CRP, AST, ALT, ALB, pH, PaCO_2_, serum sodium, serum potassium, blood lactate, blood uric acid, PT, INR, APTT, FIB, or TT upon admission (*P* > 0.05, Table 1).

### 3.3 Multivariate analysis of CMVT in elderly patients with AECOPD

A multivariate logistic regression analysis was performed using the presence or absence of CMVT as the dependent variable, and the factors showing statistical significance in the univariate analysis (Calf circumference, RBC, D-dimer, PaO_2_) as independent variables. Calf circumference was identified as a protective factor against CMVT. Conversely, RBC count and D-dimer level were identified as independent risk factors for CMVT in elderly patients with AECOPD (*P* < 0.05, Table 3).

**Table 3:** Multivariate logistic regression analysis of independent risk factors for CMVT in elderly patients with AECOPD.

Variable	B	SE	*z*	*p*	OR [95% CI]
Calf Circumference	−1.403	0.444	−3.162	0.002	0.25 [0.1, 0.59]
RBC	2.988	1.484	2.013	0.044	19.85 [1.08, 363.96]
D-dimer	0.611	0.249	2.451	0.014	1.84 [1.13, 3.01]

### 3.4 Predictive value of independent risk factors for CMVT

**Figure 2:**
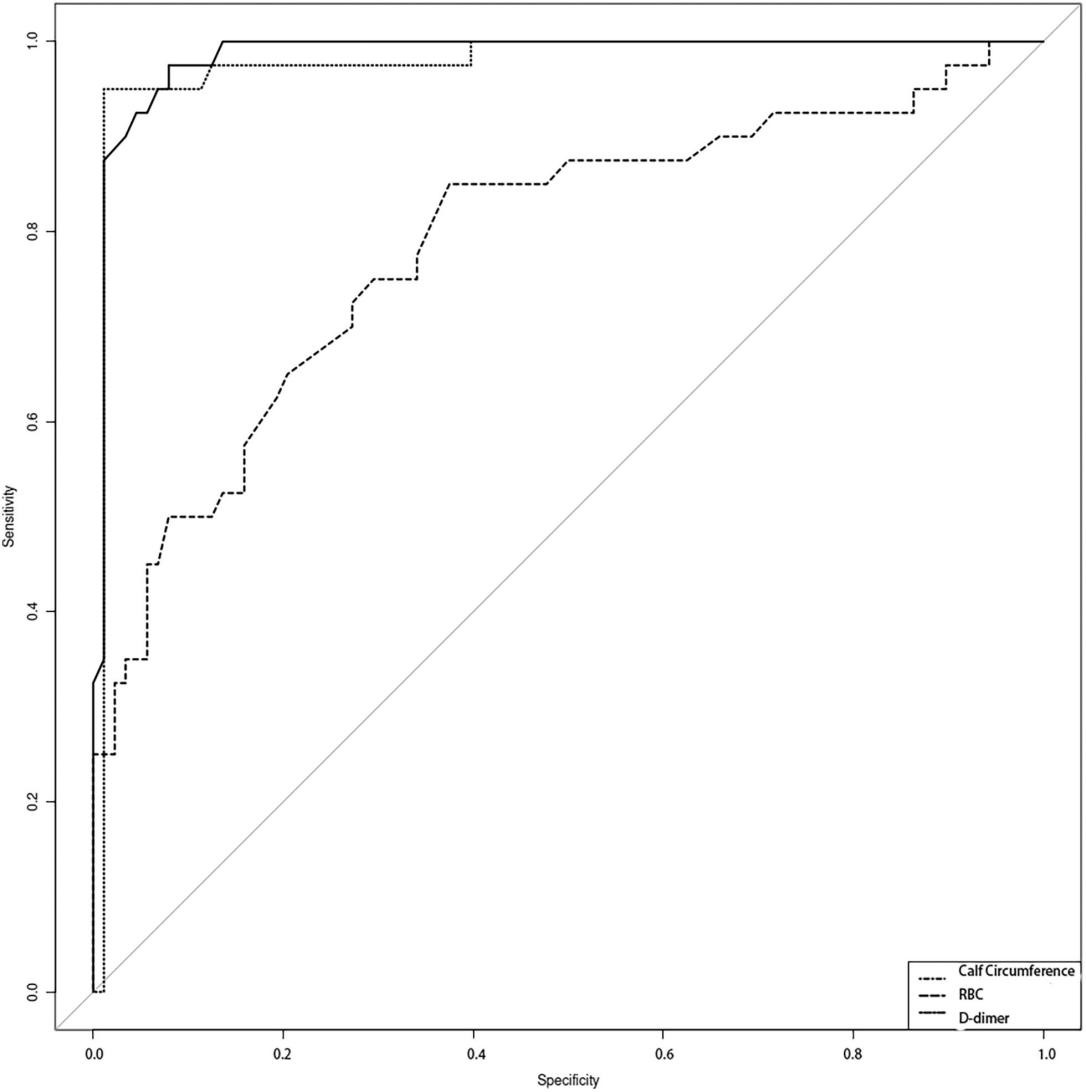
The predictive capacity of calf circumference, RBC, and D-dimer levels for chronic thromboembolism in elderly COPD patients was evaluated using receiver operating characteristic (ROC) curve analysis.

Using the presence or absence of CMVT as the dependent variable, the probability values derived from the logistic regression model for the independent risk factors (Calf circumference, RBC count, D-dimer level) were used to construct the ROC curve. For calf circumference, the AUC was 0.986 (95% CI: 0.968–1.004), sensitivity was 0.975, and specificity was 0.92. For RBC count, the AUC was 0.788 (95% CI: 0.7–0.876), sensitivity was 0.85, and specificity was 0.625. For D-dimer level, the AUC was 0.976 (95% CI: 0.947–1.005), sensitivity was 0.95, and specificity was 0.989 (Table 4 and Figure 2). The ROC analysis indicates that the model incorporating these factors exhibits good predictive performance.

**Table 4:** Predictive performance of independent risk factors for CMVT in elderly patients with AECOPD.

Variable	*n*	AUC	SE	AUC [95% CI]	Cutoff value	Sensitivity	Specificity
Calf circumference	128	0.986	0.009	[0.968, 1.004]	30.15	0.975	0.92
RBC	128	0.788	0.045	[0.7, 0.876]	4.48	0.85	0.625
D-dimer	128	0.976	0.015	[0.947, 1.005]	3.31	0.95	0.989

## 4 Discussion

COPD is a common condition that poses a severe threat to human health, not only significantly impairing quality of life but also representing one of the leading causes of mortality. Epidemiological data indicate that the prevalence of COPD among individuals aged 40 years and above in China is as high as 13.7%, corresponding to an estimated patient population of nearly 100 million, underscoring a persistently high disease burden (11). Patients with AECOPD are often confined to bed due to dyspnea and reduced exercise tolerance. Prolonged immobilization can diminish lower limb mobility, leading to venous stasis and thereby increasing susceptibility to thrombosis (12). Additionally, AECOPD is frequently complicated by hypoxia, which can elevate blood viscosity and further promote thrombus formation (13). Specifically, hypoxic stimulation induces compensatory erythropoietin (EPO) production, triggering reactive erythrocytosis, which subsequently increases blood viscosity and promotes a prothrombotic state (13). Consequently, VTE, including DVT and pulmonary embolism, is relatively common in patients with AECOPD.

Through statistical analysis of clinical and laboratory data from elderly patients with AECOPD, this study identified calf circumference, RBC count, D-dimer, and PaO_2_ as predictors for CMVT. Subsequent multivariate logistic regression analysis confirmed that calf circumference, RBC count, and D-dimer are independent risk factors for CMVT in this population. Although PaO_2_ did not emerge as an independent risk factor in the multivariate model, it remained significantly associated with CMVT risk, highlighting its clinical relevance. Established mechanistic studies have demonstrated that hypoxia can induce a hypercoagulable state by upregulation of vascular endothelial growth factor (VEGF), EPO, and hypoxia-inducible factor (HIF) (14–16). Among these, HIF may particularly promote VT via signaling pathways such as VEGF/STAT3 and NLRP3. Furthermore, HIF subunits possess immunomodulatory functions, and the NLRP3 inflammasome, induced by HIF, contributes to thrombogenesis (17, 18). The observed association between lower PaO_2_ and CMVT risk in this study is consistent with these established mechanisms.

Muscle mass progressively declines with age, a process associated with reduced activity of oxidative enzymes and decreased mitochondrial content in muscle cells. In elderly patients with COPD, factors such as chronic disease burden, systemic inflammation, and physical inactivity accelerate muscle loss, leading to a higher prevalence of sarcopenia (19, 20). Calf circumference is a simple and easily obtainable anthropometric indicator related to muscle mass. Numerous studies have associated reduced calf circumference with prolonged hospitalization, increased readmission rates, and malnutrition in patients with AECOPD (21–23). Our findings identify reduced calf circumference as an independent risk factor for CMVT. In elderly patients with AECOPD, significant atrophy of the calf muscles-clinically manifested as decreased calf circumference-compromises the function of the calf muscle pump. This impairment results in venous stasis in the lower limbs, establishing a predisposing condition for the development of CMVT (3, 24). Therefore, in elderly AECOPD patients with reduced calf circumference, clinicians should maintain a high index of suspicion for CMVT, and prompt screening via lower limb venous ultrasonography is crucial.

This retrospective study identified polycythemia (manifested as elevated RBC count) as a significant risk factor for CMVT in the elderly AECOPD population. Polycythemia is relatively common in patients with COPD, as chronic hypoxia stimulates EPO production and promotes RBC proliferation (25). In the context of CMVT development, polycythemia may exacerbate thrombosis risk through several mechanisms. First, increased RBC concentration elevates blood viscosity and reduce flow velocity in the venous system, particularly in the calf muscle regions where elderly patients with AECOPD often exhibit significant venous stasis due to limited mobility. Such hemodynamic alteration promotes endothelial activation and platelet aggregation, thereby accelerating thrombus formation (26, 27). Additionally, polycythemia may indirectly influence coagulation function by enhancing oxidative stress and inflammatory responses—both of which are markedly intensified during AECOPD and are known to increase the risk of VTE (28, 29). The interaction between chronic inflammation in COPD and polycythemia might further amplify the prothrombotic state. However, the retrospective nature of this study precludes causal inference, and potential confounding factors such as concomitant medications, comorbidities, or variations in COPD severity may have influenced these associations.

D-dimer, a fibrin degradation product, is considered to play a critical role in the thrombotic processes associated with AECOPD (30). Our study identified elevated D-dimer as an independent risk factor for CMVT in elderly patients with AECOPD. Patients with COPD often exhibit chronic inflammation, hypoxemia, and endothelial dysfunction, all of which can activate the coagulation system, leading to increased D-dimer levels. In the context of CMVT, elevated D-dimer may reflect ongoing fibrinolysis and thrombotic burden, thereby increasing the risk of venous thrombotic events (13, 30–33). Specifically, the systemic inflammatory state in COPD may promote a hypercoagulable state by upregulating pro-inflammatory cytokines (e.g., tumor necrosis factor-α and interleukin-6), which enhance thrombin generation and fibrin formation (34–36). Furthermore, chronic hypoxia may exacerbate this process by altering the fibrinolytic balance through the HIF pathway, positioning D-dimer as a potential biomarker for assessing the risk of thrombosis (37, 38).

A study by Hu et al. (39) also adopted a retrospective design to develop a prediction model for CMVT in AECOPD patients, identifying hypertension, elevated mean platelet volume (MPV), ALB, elevated D-dimer, and bed rest ≥3 days as independent risk factors. Consistent with their findings, our study also confirms that elevated D-dimer is a significant and strong independent risk factor for CMVT, further reinforcing the important role of elevated D-dimer in predicting COPD combined with CMVT. Different from the study by Hu et al., our current research focuses specifically on an elderly population (age ≥60 years). In our results, we did not find hypertension or ALB to be independent predictors. Instead, we identified reduced calf circumference and elevated RBC count as significant independent risk factors. The reason for this difference in results may be related to the different study populations selected. The elderly AECOPD patients aged 60 years and above have a higher incidence of sarcopenia and age-related muscle loss. Identifying reduced calf circumference as a protective factor against CMVT highlights the critical role of the calf muscle pump in venous return. In addition, while the Hu et al. study focused on platelet indices (MPV), our findings emphasize the hemorheological component of thrombosis risk, namely RBC count. Our results suggest that risk assessment tools may need to be tailored for specific age subgroups within the AECOPD population. Where necessary, prospective studies comparing these risk factors across different age groups should be conducted to refine prevention strategies.

It is important to emphasize that D-dimer elevation is nonspecific and may be influenced by various conditions such as infection, malignancy, or recent surgery, which are prevalent in elderly patients with AECOPD. Therefore, although this study has revealed an association between D-dimer and CMVT, the causal relationship remains unclear. D-dimer may be more a consequence rather than a direct cause of thrombosis. Nonetheless, clinicians should maintain vigilance and consider early screening for CMVT in elderly AECOPD patients with elevated D-dimer levels.

Notably, the statistical results of this study did not identify age as a significant risk factor for CMVT in this elderly AECOPD cohort, which contrasts with numerous previous studies reporting advanced age as a risk factor for VTE (40–42). This discrepancy may be attributed to the inherent age restriction of the study population (≥60 years), a design feature that may have minimized the age variation and its effect within this specific elderly cohort.

## 5 Strengths and limitations

This study has several limitations, including its retrospective, single-center design, relatively small sample size, lack of internal or external validation, and potential residual confounding from unmeasured factors (e.g., concomitant medications). Given the inherent limitations of the retrospective study design, the current findings should be considered hypothesis-generating rather than conclusive. Ideally, larger-scale, and preferably multicenter, prospective studies are warranted to validate our results. These should involve serial measurements of calf circumference, RBC, and D-dimer in elderly patients with AECOPD to enable dynamic assessment of the risk for CMVT.

## 6 Conclusion

In conclusion, this study confirms that reduced calf circumference, elevated RBC count, and elevated D-dimer levels are independent risk factors for the development of CMVT in elderly patients with AECOPD. Consequently, clinicians should closely monitor these indicators during the diagnosis and treatment of such patients to achieve early prevention and detection of CMVT.

## Data Availability

The original contributions presented in the study are included in the article/Supplementary Material, further inquiries can be directed to the corresponding author.
